# Health Care Access Measures and Palliative Care Use by Race/Ethnicity among Metastatic Gynecological Cancer Patients in the United States

**DOI:** 10.3390/ijerph18116040

**Published:** 2021-06-04

**Authors:** Jessica Y. Islam, Veeral Saraiya, Rebecca A. Previs, Tomi Akinyemiju

**Affiliations:** 1 Lineberger Comprehensive Cancer Center, University of North Carolina at Chapel Hill, Chapel Hill, NC 27514, USA; islamjy@email.unc.edu; 2 Cancer Epidemiology Program, Center for Immunization and Infection Research in Cancer (CIIRC), H. Lee Moffitt Cancer Center and Research Institute, Tampa, FL 33612, USA; 3 Department of Population Health Sciences, Duke University School of Medicine, Durham, NC 27701, USA; 4 Department of Epidemiology, University of North Carolina Gillings School of Global Public Health, Chapel Hill, NC 27514, USA; veeral@unc.edu; 5 Division of Gynecological Oncology, Duke Cancer Institute, Durham, NC 27710, USA; Rebecca.previs@duke.edu

**Keywords:** racial disparities, gynecologic cancers, social determinants of health, health care access, insurance type, distance-to-care, gynecological malignancies

## Abstract

Palliative care improves quality-of-life and extends survival, however, is underutilized among gynecological cancer patients in the United States (U.S.). Our objective was to evaluate associations between healthcare access (HCA) measures and palliative care utilization among U.S. gynecological cancer patients overall and by race/ethnicity. We used 2004–2016 data from the U.S. National Cancer Database and included patients with metastatic (stage III–IV at-diagnosis) ovarian, cervical, and uterine cancer (n = 176,899). Palliative care was defined as non-curative treatment and could include surgery, radiation, chemotherapy, and pain management, or any combination. HCA measures included insurance type, area-level socioeconomic measures, distance-to-care, and cancer treatment facility type. We evaluated associations of HCA measures with palliative care use overall and by race/ethnicity using multivariable logistic regression. Our population was mostly non-Hispanic White (72%), had ovarian cancer (72%), and 24% survived <6 months. Five percent of metastatic gynecological cancer patients utilized palliative care. Compared to those with private insurance, uninsured patients with ovarian (aOR: 1.80,95% CI: 1.53–2.12), and cervical (aOR: 1.45,95% CI: 1.26–1.67) cancer were more likely to use palliative care. Patients with ovarian (aOR: 0.58,95% CI: 0.48–0.70) or cervical cancer (aOR: 0.74,95% CI: 0.60–0.88) who reside >45 miles from their provider were less likely to utilize palliative care than those within <2 miles. Ovarian cancer patients treated at academic/research programs were less likely to utilize palliative care compared to those treated at community cancer programs (aOR: 0.70, 95%CI: 0.58–0.84). Associations between HCA measures and palliative care utilization were largely consistent across U.S. racial-ethnic groups. Insurance type, cancer treatment facility type, and distance-to-care may influence palliative care use among metastatic gynecological cancer patients in the U.S.

## 1. Introduction

Palliative care is an integral aspect of high-quality cancer treatment. The United States (U.S.) National Comprehensive Cancer Network (NCCN) recommends palliative care should begin at cancer diagnosis and be delivered concurrently with regular cancer life-prolonging treatment to relieve symptom burden [1]. Palliative care can improve health-related quality of life by addressing symptoms frequently experienced during cancer treatment, such as pain, nausea, fatigue, neuropathy, as well as psychosocial symptoms [2]. Due to its documented benefits in symptom relief and improvements in survival among patients with advanced cancer [3,4,5,6,7,8], palliative care is also recommended by the American Society of Clinical Oncology (ASCO) and the Society of Gynecologic Oncology (SGO) throughout the cancer care continuum [1,9,10].

Palliative care is underutilized among U.S. patients with gynecological cancers despite the high symptom burden during treatment for metastatic disease (stage III-IV cancer at diagnosis) [11,12,13,14]. For example, in a retrospective study of deceased patients with ovarian cancer, only 28% were referred to palliative care, and the most common type of palliative care was a referral to hospice care rather than palliation of adverse symptoms [13]. Reasons for the underutilization of palliative care among U.S. gynecological cancer patients remain unclear [15]. Additionally, existing U.S. studies have focused on palliative care during the end-of-life setting rather than across the cancer care continuum.

Several factors associated with the use of palliation in the context of end-of-life care among cancer patients have been identified, including differences by race/ethnicity. Racial minorities are less likely to utilize hospice services at end-of-life compared to their White counterparts, and these differences have been attributed to knowledge gaps and attitudes among patients and providers alike [16,17]. In contrast, however, Black gynecologic cancer patients have been found to be more likely to utilize inpatient palliative care services before death compared to non-Hispanic White gynecologic cancer patients [18]. Near end-of-life, Medicaid-insured and uninsured gynecological cancer have been found to be more likely to utilize palliative care compared to those with Medicare insurance [18]. Other measures related to health care access, such as distance from provider to patient, have not been evaluated among metastatic gynecological cancer patients in the palliative care context. 

In our prior work, we demonstrated that racial/ethnic disparities exist in palliative care use among metastatic gynecological patients, specifically that NH-Black and Hispanic patients are less likely to use palliative care compared to their NH-White counterparts in the U.S. [14,19]. A current knowledge gap exists regarding the role of measures of health care access with palliative intervention use among metastatic gynecological cancer patients, and in particular, whether healthcare access factors may explain any observed racial/ethnic differences in palliative care utilization in the U.S. The objective of this study was to evaluate the following health care access measures: patient’s insurance status, Medicaid expansion status of the state the patient was treated, distance from patient-to-provider, cancer treatment facility type, percent of adults without a high school degree in patient’s zip code (a surrogate measure for educational level), and median household income of adults in patient’s zip code (a surrogate measure for income level). We evaluated associations of palliative care utilization with these health care access measures by metastatic gynecological cancer site of origin overall and by race/ethnicity. We hypothesized that measures of high health care access (i.e., private insurance; receiving treatment at an academic medical center; residing in a highly educated area, etc.,), will be positively associated with using palliative care. 

## 2. Materials and Methods

### 2.1. Data Source

Data for this study were obtained from the latest 2004–2016 Participant Use Files (PUF) of the U.S. National Cancer Data Base (NCDB), a United States hospital-based oncology database combining data on patients seen at any of the 1500 Commission on Cancer (CoC) accredited institutions in the United States [20,21]. The NCDB registry is a joint project of the American Cancer Society and the Commission on Cancer of the American College of Surgeons. The NCDB registry includes more than 29 million unique cases or 70% of all patients with newly diagnosed cancer in the United States [22]. Data reported to the NCDB are highly standardized similar to other state health departments and federal cancer registry data systems, including the U.S. Surveillance, Epidemiology, and End Results (SEER). Data included in the NCDB are from patient charts abstracted by Certified Tumor Registrars (CTR) who undergo training specific to cancer registry operations [23]. The data abstractors use standardized methods to collect sociodemographic, including race/ethnicity, and clinical data, including tumor type, stage, grade, and treatments. To ensure high-quality and accurate data, CoC-accredited sites undergo an external review of hospital charts and registry abstracts to verify the NCDB registry data correctly reflect the information documented in individual patient records using a sample of at least 10% of records [24]. The study was approved by Duke University Institutional Review Board (Durham, NC, USA) under a general study protocol (IRB#: Pro00102834). 

### 2.2. Study Cohort

Study participants included patients with Stage-III and IV ovarian, cervical, and uterine cancers at diagnosis. We included patients diagnosed between 1 January 2004 to 31 December 2016 using the following International Classification of Diseases for Oncology, Third Edition topography codes: ovarian (C569), cervical (C530, C531, C538, C539), and uterine (C559). Patients with missing or unknown cancer stages were excluded (n = 35,346, 9.9%). We excluded patients with missing data on palliative care utilization (n = 1018, 0.6%). Overall, our study population included 176,899 patients. In sensitivity analyses, we excluded metastatic gynecological cancer patients who were known to be alive based on the vital status of the patient as of the last date of contact (n = 52,170, 32.2%). We evaluated deceased metastatic gynecological cancer patients (n = 124,729) as palliative care has historically been prioritized among those near the end-of-life. Results based on sensitivity analyses are summarized in Supplementary Material. 

### 2.3. Palliative Care Utilization

The main outcome was palliative care as defined by the NCDB, as in previously published studies [13,21,25,26,27]. The NCDB includes information on any palliative care from patients’ clinical medical records during their treatment at the reporting facility. The NCDB codes treatments as palliative only if the patient’s medical records explicitly mentioned that the goal of treatment is palliation and not cure. Specifically, any procedure was categorized as palliative care if treatment was provided to “prolong a patient’s life by controlling symptoms, to alleviate pain, or to make the patient more comfortable [28].” Types of palliative care documented and abstracted from the patient’s medical record could include pain management therapy, surgery, radiation therapy, or systemic chemotherapy administered to alleviate symptoms. Patients utilizing palliative care in the NCDB may also concurrently be undergoing curative treatment. The NCDB does not document hospice services or referral to hospice and was therefore not included in the definition of palliative care. Palliative care utilization was compared to those who did not utilize palliative care.

### 2.4. Study Variables

We evaluated several health care access variables, which we define here. Insurance type was identified as the patient’s primary insurance carrier at the time of initial diagnosis and/or treatment. Types of insurance include private insurance, which is traditionally provided by the patient’s employer or union, Medicare, Medicaid, and other Government insurance. Medicaid and Medicare are U.S. government-sponsored national health insurance programs; Medicaid is available to eligible low-income adults, children, pregnant women, elderly adults, and people with disabilities [29]. The Medicare program is a federal health insurance program for people who are 65 years or older, certain younger people with disabilities, and people with end-stage renal disease [30]. Other government insurance may include the Indian Health Services Insurance, which is offered to U.S. adults who identify as Native Americans; the Veterans Health Administration or the VA, which is offered to U.S. adults who have served the U.S. military. Also, we evaluated the potential association of Medicaid expansion status of the state the patient was diagnosed. In the U.S., states have an option to expand the eligibility requirements to enroll in Medicaid to cover more low-income Americans [31]. As of 2019, Medicaid has fully expanded in 33 states and the District of Columbia. It is important to evaluate the impact of Medicaid expansion on the use of health services in the U.S. to provide evidence of the benefits of the program and the potential long-term cost savings after investment into the program developed by the Affordable Care Act (ACA). We only included data starting from 1 January 2011 to 31 December 2016 as the Affordable Care Act was passed in 2010 and enacted in the following year [32]. We evaluated the greatest circle distance from provider to patient, which is a measure of distance in miles between the patient’s residence (residential latitude and longitude based on the patient’s zip code centroid or the city if the zip code was not available) and the provider’s hospital location (street address for the facility). Additionally, we evaluated the percentage of adults in the patient’s zip code without a high school degree and median household income in the patient’s zip code. These zip code level variables were derived from the 2012 American Community Survey (ACS) data, spanning years 2008–2012, and adjusted for 2012 inflation [33]. The ACS is an ongoing survey conducted by the U.S. Census Bureau that provides annual demographic data on U.S. communities. Further details regarding the ACS can be found here: https://www.census.gov/programs-surveys/acs/about.html (accessed on 25 May 2021). The percentage of adults with a high school degree and median household income was categorized as quartiles based on equally proportioned income ranges among all United States zip codes. 

Race/ethnicity, as defined by the U.S. Census Bureau [34,35], were captured in the NCDB based on self-report or as reported by the patient’s providers. In the U.S., ethnicity is defined as Hispanic or Latino, and Not Hispanic or Latino. The U.S. Census Bureau defines “Hispanic or Latino” as a person of Cuban, Mexican, Puerto Rican, South or Central American, or other Spanish culture or origin regardless of race [35]. U.S.-specific definitions of racial categories can be found here: https://www.census.gov/topics/population/race/about.html (accessed on 25 May 2021). We evaluated the health care access variables overall and stratified by race/ethnicity. We combined reported race/ethnicity to create the following categories: Non-Hispanic (NH) White (NH-White), NH-Black, Hispanic, Asian, American Indian/Alaskan Native, Native Hawaiian/Pacific Islander, and other Race. For the main analysis, we focused on comparisons of NH-White, NH-Black, Hispanic, and Asian metastatic gynecological cancer patients to ensure adequate patient size across racial groups for statistical modeling. 

### 2.5. Statistical Analysis

We summarized patient characteristics as percentages by palliative care utilization among metastatic (Stage III/IV at diagnosis) ovarian, cervical, or uterine cancer patients at the time of presentation. We evaluated racial/ethnic differences in health care access measures using bivariate statistical analyses (χ^2^ tests). Next, we evaluated multivariable associations of health care access measures stratified by cancer site and next by race/ethnicity using multivariable logistic regression. We estimated adjusted odds ratios and 95% confidence intervals and adjusted for study covariates identified using directed acyclic graphs based on prior literature for health care access and palliative care. Adjustment sets for each health care access measure are summarized in Tables accordingly. We accounted for non-independence within clusters at the facility level to account for correlated patient characteristics within hospitals and calculated cluster-robust standard errors. We assessed each covariate for collinearity and used a complete case approach. All analyses were performed with Stata statistical software, version 15.0 (StataCorp, College Station, TX, USA).

## 3. Results

Overall, the median age of patients was 62 years, and most were non-Hispanic White (72%) and either insured with Medicare (41%) or privately insured (36%). Twenty-one percent of patients had a Charlson-Deyo comorbidity score of one or above. Most lived in urban areas (96%) and were treated at either a comprehensive community cancer program (36%) or an academic/research program (38%). Five percent of patients with metastatic gynecological cancer at the time of presentation utilized palliative care at any time during their disease course. Overall, 4% of patients with ovarian, 9% with cervical, and 11% with uterine metastatic cancer utilized palliative care. Among patients who did receive palliative, the most common types included either surgery, radiation, or chemotherapy alone (62%) and 12% received pain management only (Table 1). 

Table 2 summarizes each health care access measure stratified by race/ethnicity. NH-White (86%) and NH-Black (69%) patients were mostly either privately or Medicare insured. Asian patients commonly lived in the Western census region (43%), whereas NH-Black patients commonly resided in the South (53%) (*p* < 0.001). We observed the highest proportion of Hispanic patients (58%) residing in zip codes with ≥17.6% of adults without a high school degree (i.e., less educated), and the lowest among NH-White patients (16%) (*p* < 0.001). Patients were commonly treated at academic/research programs, particularly NH-Black (46%), Hispanic (43%), and Asian (45%) patients. 

Compared to privately insured patients, uninsured patients with ovarian (aOR: 1.80, 95% CI: 1.53–2.12) and cervical cancer (aOR: 1.45, 95% CI: 1.26–1.67) were more likely to utilize palliative care after adjustment for age, race/ethnicity, Charlson-Deyo comorbidity score, and median household income (Table 3). Medicaid-insured patients with ovarian cancer (aOR: 1.89, 95% CI: 1.64–2.19) and cervical cancer (aOR: 1.41, 95% CI: 1.26–1.57) were more likely to utilize palliative care. When evaluating the greatest circle distance from provider to patient, we observed that compared to patients <2 miles away from their provider, the odds of utilizing palliative care decreased with increasing distance for all gynecological cancer sites after adjustment for age, urban or rural area of residence, and census region. Compared to ovarian cancer patients who were treated at comprehensive community cancer programs, academic/research program patients were less likely to utilize palliative care (aOR: 0.70, 95% CI: 0.58–0.84). Sensitivity analyses revealed similar patterns of palliative care utilization across cancer sites for each health care access measure among deceased metastatic gynecologic cancer patients (Table S1). 

**Figure 1:**
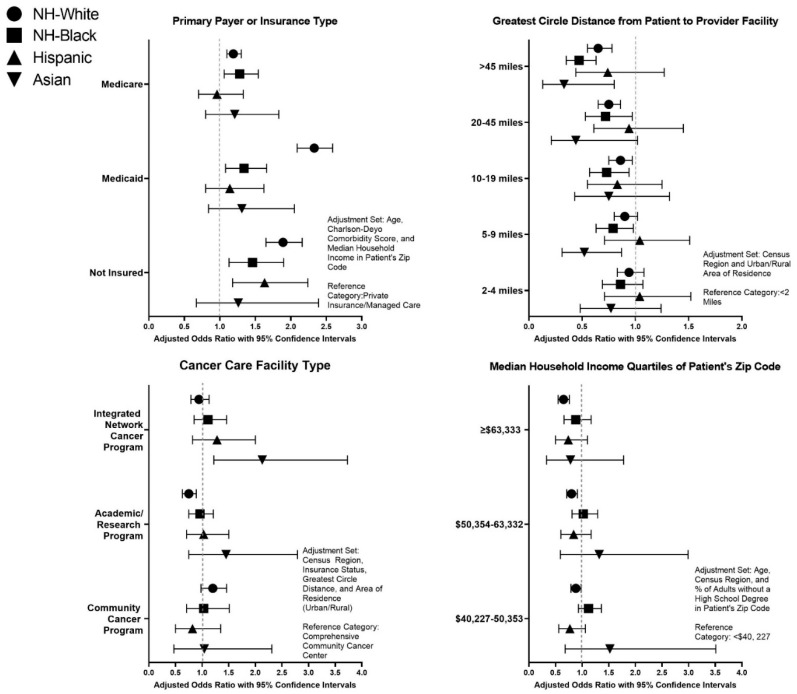
Associations of Health Care Access Measures with Palliative Care Use Among Metastatic Gynecological Cancer Patients by Race/Ethnicity.

Figure 1 provides a summary of health care access measures evaluated by race/ethnicity by gynecological cancer sites, and the point estimates are available in Table S2. Uninsured NH-White (aOR: 1.98, 95% CI: 1.74–2.24), NH-Black (aOR: 1.47, 95% CI: 1.18–1.83), and Hispanic (aOR: 1.86, 95% CI: 1.38–2.51) patients were more likely to utilize palliative care when compared to patients with private insurance. NH-White and NH-Black patients with Medicaid or Medicare were more likely to use palliative care compared to their privately insured counterparts. Asian patients residing in more educated areas were more likely to use palliative; For example, compared to Asian patients residing in areas with ≥17.6% of adults without a high school degree (i.e., least educated), Asian patients in the most educated areas (<6.3% of adults without a high school degree) had over two times the odds of using palliative care (aOR: 2.41, 95% CI: 1.43–4.07). Compared to NH-White, NH-Black, and Asian patients residing <2-mile circle distance away from their provider, those who reside more than 45 miles away had 37% (aOR: 0.63, 95% CI: 0.53–0.74), 54% (aOR: 0.46, 95% CI: 0.35–0.61), and 66% (aOR: 0.34, 95% CI: 0.14–0.83) lower odds of palliative care utilization, respectively. NH-White (aOR: 0.73, 95% CI: 0.62–0.86) patients treated at academic/research programs were less likely to utilize palliative care compared to those treated at comprehensive community cancer programs. Conversely, Asian (aOR: 2.01, 95% CI: 1.15–3.51) patients treated at Integrated Network Cancer Programs were more likely to utilize palliative care. Increasing distance from patient to provider also led to lower odds of palliative care, utilization specifically among NH-White, NH-Black, and Asian patients. Sensitivity analyses revealed similar findings among deceased metastatic gynecologic cancer patients across racial/ethnic groups (Table S3). 

## 4. Discussion

In our study of metastatic gynecological cancer patients treated at Commission on Cancer (CoC) accredited institutions in the United States, we observed that several measures of health care access were important predictors of palliative care use. Distance-to-care plays an important role in palliative care use among patients with metastatic gynecological cancers: Patients living farther from their providers were less likely to utilize palliative care than those living closer to their provider, and this trend was consistent across racial groups. Uninsured patients and patients with Medicaid or Medicare insurance were more likely to utilize palliative care compared to the privately insured, particularly patients with ovarian or cervical cancer. Understanding health care access measures that influence palliative care use may reveal areas for intervention to improve access to equitable high-quality cancer care among gynecologic cancer patients in the U.S. and globally, where patients may experience similar barriers to palliative care use.

Our study suggests that patients living farther from their providers were less likely to utilize palliative care, and this finding was consistent across racial groups. Our finding is similar to prior studies demonstrating distance-to-care impacts high-quality cancer care, including studies evaluating cancer treatment outcomes such as receipt of guideline adherent care [36]. For example, a prior study found that urban women receiving curative treatment for cervical cancer who lived farther than 15 miles away from their provider were less likely to initiate timely treatment compared with those <5 miles from their provider [37]. Prior work evaluating distance-to-care using the NCDB has also reported similar findings: for example, stage III colon cancer patients who traveled 50 to 249 miles for treatment were less likely to receive adjuvant chemotherapy than patients with a travel distance less than 12.5 miles [38]. Limited studies have evaluated distance-to-care in the palliative care context. The disparity we observed may be attributable to geographic disparities that exist in the distribution of gynecologic oncologists across the United States [39]. A survey of gynecologic oncologists showed that almost three-quarters practiced in an urban setting and only 13% practiced in an area with a population <50,000, i.e., rural areas [39]. Patients living farther from their cancer provider may be more likely to live in a rural community and therefore receive cancer treatment at a smaller community hospital. Smaller community hospitals may be less likely to have palliative care programs in place, leading to the underutilization of palliative care services [40]. Future research evaluating rural and urban differences in palliative care use among cancer patients should be prioritized to optimize rural cancer care. 

The NCDB provides a unique opportunity to evaluate the role of health care access from the perspective of several types of insurance providers. Our work demonstrates patient’s insurance type plays an important role in palliative care use. We observed that patients with ovarian and cervical cancer insured through Medicaid and patients without health insurance were more likely to utilize palliative care compared to those on private insurance. Our finding is consistent with a prior NCDB study of patients with colon, lung, melanoma, and prostate cancer patients, which also demonstrated that Medicaid insurance was a determinant of increased palliative care use when compared to privately insured patients [41]. In the U.S, patients insured through Medicaid or without health insurance are more likely to be low-income or without employment, which in turn leads to poor access to U.S. health care due to the prohibitive costs associated with cancer treatment in the U.S. As such, uninsured or Medicaid-insured patients are less likely to access care at early stages of cancer development leading to metastatic cancer at diagnosis. Prior research shows that palliative care is prioritized near the end-of-life or when cancer has progressed, which may explain this finding. Further, previous research has shown that the delivery of palliative care services to individuals with Medicaid insurance results in lower healthcare costs to the hospital and providers, especially when treatment was delivered to patients at the end of life and died due to their cancer [42,43,44]. These cost savings associated with palliative care services provided to Medicaid patients may also explain our results, however, further research is needed to evaluate the impact of insurance type. 

Metastatic gynecologic cancer patients receiving care at academic, or research hospitals were less likely to receive palliative care compared to comprehensive community cancer programs. Also, our data suggest ovarian cancer patients receiving care through community cancer programs may be more likely to use palliative care, particularly those who may have passed due to their cancer. Our finding is in contrast to prior work that found public hospitals, sole community provider hospitals, and for-profit hospitals are less likely to have palliative programs and services compared to hospitals affiliated with medical schools or large hospitals [45]. However, this prior work explored the broader question of the existence of palliative care programs generally while we evaluated patient-level utilization of the palliative care programs specifically among gynecologic cancer patients. Our findings are also in contrast to a prior NCDB study which showed that colorectal cancer patients receiving care at academic or research programs were more likely to utilize palliative care when compared to non-academic programs, which is in contrast to our comparison group and potentially contributing to the different findings [27]. Further research is needed to delineate differences in palliative care use by cancer care facility types in the United States.

The use of palliative care is influenced by several factors, including patient characteristics, disease characteristics, and provider characteristics. We are limited to the data available in the NCDB and are not able to evaluate unmeasured factors that may influence the choice of physician or patient to opt for palliative care. For example, patients may not receive palliative care due to personal choice or beliefs regarding end-of-life care [46]. Additionally, further investigation of the role of area-level societal factors that may play a role in access to care at the patient level will be an important area of research to deliver equitable cancer care in the U.S. This analysis was limited to zip-code level proxy measures for educational attainment and income level. However, prior research has demonstrated the limitations of leveraging zip-code level measures as a proxy for individual level socioeconomic status [47]. Future research should prioritize leveraging a more precise measure of area-level socioeconomic status, such as census-tract level measures. It is also important to acknowledge the NCDB data on palliative care services are of uncertain accuracy. Palliative intent must be inferred from clinical records, and therefore, there is an opportunity for misclassification of palliative care use and the type of palliative care treatment. However, the NCDB has established protocols to ensure the data are captured accurately and as noted in prior work, record abstraction methods used to develop the NCDB is the approach leveraged by all hospital-based studies evaluating palliative care and is likely to be more accurate than health insurance claims. 

## 5. Conclusions

We explored health care access measures to inform the under-utilization of palliative care services among patients with metastatic gynecological cancers in the United States. We observed that patients who had Medicaid or who were uninsured were more likely to use palliative care, that individuals living far away from their provider were less likely to receive palliative care, and that individuals receiving care at academic, or research hospitals were less likely to receive palliative care compared to the referent group. Our results suggest racial and ethnic identities may play an important factor in palliative care utilization among women with metastatic gynecological cancers, potentially due to structural barriers racial minorities experience to obtain high-quality cancer care: Limitations may exist in the race/ethnicity data captured in the NCDB due to differences in race recording practices across participating institutions. For example, some institutions may rely on an individual’s last name to assign Hispanic ethnicity, which is an unreliable measure of racial/ethnic identity. Future research conducted to evaluate racial/ethnic disparities in palliative care use should prioritize efforts to optimize the capture of self-defined racial/ethnic identity. Also, given the differences in the demographic composition of patient populations by cancer type, future research should investigate the role of health care access factors in palliative care use in the context of other cancer sites to improve uptake and accessibility of palliative care for all cancer patients. Equitable access to palliative care is an important metric of high-quality cancer care in the United States, and efforts to improve the delivery of palliative care services using insights from our analysis should be prioritized. 

## Figures and Tables

**Table 1:** Health Care Access Measures Among Metastatic Gynecologic Cancer Patients by Palliative Care Utilization (n = 176,899).

	Total (n = 176,899)		No Palliative Care (n = 167,071)		Palliative Care Used (n = 9282)	
	No.	Col %	No.	Row %	No.	Row %
Age (Mean, SD)	62.1, 14.3		61.8, 14.3		65.5, 14.7	
Cancer Type						
Ovarian cancer	127,237	71.9	121,767	95.7	5470	4.3
Cervical cancer	42,944	24.3	39,298	91.5	3646	8.5
Uterine cancer	6718	3.8	6006	89.4	712	10.6
Race/Ethnicity						
Non-Hispanic White	128,096	72.4	120,970	94.4	7126	5.6
Non-Hispanic Black	19,259	10.9	17,905	93.0	1354	7.0
Hispanic	12,790	7.2	12,231	95.6	559	4.4
Asian	5066	2.9	4839	95.5	227	4.5
American Indian/Alaskan Native	683	0.4	650	95.2	33	4.8
Native Hawaiian/Pacific Islander	367	0.2	332	90.5	35	9.5
Other Race	9561	5.4	9107	95.3	454	4.7
Missing	1077	0.6	1037	96.3	40	3.7
Palliative care provided (Col %)						
No palliative care	167,071	94.4	167,071	100	0	0
Surgery/radiation/chemo only	6101	3.4	0	0	6101	62.1
Pain management only	1198	0.7	0	0	1198	12.2
Combination of surg/rad/chemo and pain management	1027	0.6	0	0	1027	10.4
Type unknown	1502	0.8	0	0	1502	15.3
Charlson-Deyo Score (Comorbidities)						
0	140,231	79.3	133,103	94.9	7128	5.1
1	27,557	15.6	25,715	93.3	1842	6.7
2	6393	3.6	5823	91.1	570	8.9
≥3	2718	1.5	2430	89.4	288	10.6
Primary Payer						
Not Insured	9689	5.5	9055	93.5	634	6.5
Private Insurance/Managed Care	70,522	39.9	67,846	96.2	2676	3.8
Medicaid	18,784	10.6	17,463	93.0	1321	7.0
Medicare	73,151	41.4	68,174	93.2	4977	6.8
Other Government	1679	0.9	1595	95.0	84	5.0
Insurance Status Unknown	3074	1.7	2938	95.6	136	4.4
Census Region						
Northeast	34,237	19.4	31,674	92.5	2563	7.5
South	62,447	35.3	59,039	94.5	3408	5.5
Midwest	40,047	22.6	37,689	94.1	2358	5.9
West	28,517	16.1	27,451	96.3	1066	3.7
Missing	11,651	6.6	11,218	96.3	433	3.7
Area of Residence						
Urban	169,092	95.6	159,674	94.4	9418	5.6
Rural	3040	1.7	2854	93.9	186	6.1
Missing	4767	2.7	4543	95.3	224	4.7
Percent of Adults in Patient Zip Code with No High School Degree Quartiles 2012–2016						
≥17.6%	39,998	22.6	37,767	94.4	2231	5.6
10.9–17.5%	46,017	26.0	43,265	94.0	2752	6.0
6.3–10.8%	47,877	27.1	45,229	94.5	2648	5.5
<6.3%	40,455	22.9	38,383	94.9	2072	5.1
Missing	2552	1.4	2427	95.1	125	4.9
Median Household Income Quartiles of Patients in Zip Code 2012–2016						
<$40,227	35,225	19.9	32,983	93.6	2242	6.4
$40,227–50,353	39,605	22.4	37,229	94.0	2376	6.0
$50,354–63,332	40,619	23.0	38,378	94.5	2241	5.5
≥$63,333	58,587	33.1	55,767	95.2	2820	4.8
Missing	2863	1.6	2714	94.8	149	5.2
Patient State at Diagnosis Grouped by Medicaid Expansion Status 2010–2016 *						
Non-Expansion States	31562	35.6	29,497	93.5	2065	6.5
January 2014 Expansion States	24,922	28.1	22,909	91.9	2013	8.1
Early Expansion States (2010–2013)	15,408	17.4	14,713	95.5	695	4.5
Late Expansion States (after Jan. 2014)	10,767	12.2	9,849	91.5	918	8.5
Suppressed for Ages 0–39	5,978	6.7	5,714	95.6	264	4.4
Greatest Circle Distance from Provider to Patient						
<2 miles	15,273	8.6	14,187	92.9	1086	7.1
2–4 miles	30,521	17.3	28,551	93.5	1970	6.5
5–9 miles	36,155	20.4	34,003	94.0	2152	6.0
10–19 miles	34,836	19.7	32,926	94.5	1910	5.5
20–45 miles	31,500	17.8	29,963	95.1	1537	4.9
>45 miles	28,614	16.2	27,441	95.9	1173	4.1
Facility Type						
Community Cancer Program	10,475	5.9	9660	92.2	815	7.8
Comprehensive Community Cancer Program	63,663	36.0	59,851	94.0	3812	6.0
Academic/Research Program	68,317	38.6	64,939	95.1	3378	4.9
Integrated Network Cancer Program	22,793	12.9	21,403	93.9	1390	6.1
Missing	11,651	6.6	11,218	96.3	433	3.7

Abbreviations: No.: Number; Row %: Row percentages; Col %: Column percentages; SD: Standard Deviation. * Data were restricted to 2011–2016 as the Affordable Care Act was passed in 2010 and enacted in the following year (n = 88,637).

**Table 2:** Health Care Access Measures Among Metastatic Gynecological Cancer Patients by Race/Ethnicity (n = 165,211) *.

	NH-White		NH-Black		Hispanic		Asian		p †
	n	Col %	n	Col %	n	Col %	n	Col %	
Primary Payer									<0.001
Not Insured	5008	3.9	1618	8.4	2122	16.6	385	7.6	
Private Insurance/Managed Care	53,177	41.5	6247	32.4	3983	31.1	2372	46.8	
Medicaid	9997	7.8	3697	19.2	3117	24.4	833	16.4	
Medicare	56,933	44.4	7056	36.6	3145	24.6	1278	25.2	
Other Government	1141	0.9	173	0.9	91	0.7	92	1.8	
Insurance Status Unknown	1840	1.4	468	2.4	332	2.6	106	2.1	
Census Region									<0.001
Northeast	25,725	20.1	3294	17.1	1864	14.6	1033	20.4	
South	44,083	34.4	10,136	52.6	4221	33.0	918	18.1	
Midwest	31,218	24.4	3177	16.5	861	6.7	518	10.2	
West	20,116	15.7	1002	5.2	3945	30.8	2183	43.1	
Missing	6954	5.4	1650	8.6	1899	14.8	414	8.2	
Area of Residence									<0.001
Urban	121,835	95.1	18,695	97.1	12,499	97.7	4929	97.3	
Rural	2472	1.9	215	1.1	35	0.3	5	0.1	
Missing	3789	3.0	349	1.8	256	2.0	132	2.6	
Percent of Adults in Patient Zip Code with No High School Degree Quartiles 2012–2016									<0.001
≥17.6%	20,705	16.2	8213	42.6	7431	58.1	1402	27.7	
10.9–17.5%	33,189	25.9	6193	32.2	2440	19.1	997	19.7	
6.3–10.8%	38,288	29.9	3144	16.3	1688	13.2	1316	26.0	
<6.3%	34,060	26.6	1380	7.2	1060	8.3	1307	25.8	
Missing	1854	1.4	329	1.7	171	1.3	44	0.9	
Median Household Income Quartiles of Patients in Zip Code 2012–2016									<0.001
<$40,227	19,422	15.2	9026	46.9	3805	29.7	468	9.2	
$40,227–50,353	29,010	22.6	3910	20.3	3075	24.0	737	14.5	
$50,354–63,332	31,197	24.4	2777	14.4	2858	22.3	1114	22.0	
≥$63,333	46,363	36.2	3196	16.6	2871	22.4	2701	53.3	
Missing	2104	1.6	350	1.8	181	1.4	46	0.9	
Patient State at Diagnosis Grouped by Medicaid Expansion Status 2010–2016 ‡									<0.001
Non-Expansion States	22,837	35.4	4677	44.6	2401	33.9	520	17.6	
January 2014 Expansion States	19,057	29.5	2590	24.7	1359	19.2	857	28.9	
Early Expansion States (2010–2013)	10,457	16.2	1164	11.1	2076	29.4	1202	40.6	
Late Expansion States (after Jan. 2014)	8666	13.4	1181	11.3	187	2.7	148	4.9	
Suppressed for Ages 0–39	3560	5.5	879	8.4	1042	14.8	236	7.8	
Greatest Circle Distance from Provider to Patient									<0.001
<2 miles	10,229	8.0	2362	12.3	1258	9.8	517	10.2	
2–4 miles	19,986	15.6	4683	24.3	2786	21.8	1197	23.6	
5–9 miles	24,300	19.0	4843	25.1	3377	26.4	1419	28.0	
10–19 miles	25,653	20.0	3041	15.8	2654	20.8	1099	21.7	
20–45 miles	25,017	19.5	2330	12.1	1480	11.6	516	10.2	
>45 miles	22,911	17.9	2000	10.4	1235	9.7	318	6.3	
Facility Type									<0.001
Community Cancer Program	7843	6.1	979	5.1	669	5.2	378	7.5	
Comprehensive Community Cancer Program	49,259	38.5	5259	27.3	3396	26.6	1508	29.8	
Academic/Research Program	47,095	36.8	8936	46.4	5510	43.1	2301	45.4	
Integrated Network Cancer Program	16,945	13.2	2435	12.6	1316	10.3	465	9.2	
Missing	6954	5.4	1650	8.6	1899	14.8	414	8.2	

* Excludes metastatic gynecological cancer patients of other races (n = 11688). † χ-squared test *p*-value to test differences across racial/ethnic categories. ‡ Data were restricted to 2011–2016 as the Affordable Care Act was passed in 2010 and enacted in the following year (n = 88,637).

**Table 3:** Associations of Health Care Access Factors with Palliative Care Use Among All Metastatic Gynecological Cancer Patients by Cancer Site (n = 176,899).

	Ovarian Cancer (n= 127,237)			Cervical Cancer (n = 42,944)			Uterine Cancer (n = 6718)		
	% Utilized Palliative Care	aOR	95% CI	% Utilized Palliative Care	aOR	95% CI	% Utilized Palliative Care	aOR	95% CI
Primary Payer *									
Not Insured	4.2	1.80	1.53–2.12	8.6	1.45	1.26–1.67	11.6	1.19	0.81–1.73
Private Insurance/Managed Care	2.7	Ref.		6.5	Ref.		9.6	Ref.	
Medicaid	4.9	1.89	1.64–2.19	8.4	1.41	1.26–1.57	11.3	1.25	0.93–1.69
Medicare	5.7	1.19	1.09–1.31	12.1	1.14	1.02–1.27	11.1	1.15	0.92–1.43
Other Government	4.3	1.57	1.14–2.19	6.4	0.98	0.67–1.46	8.9	1.08	0.43–2.75
Percent of Adults in Patient Zip Code with No High School Degree Quartiles 2012–2016 †									
≥17.6%	3.9	Ref.		7.9	Ref.		10.8	Ref.	
10.9–17.5%	4.6	1.18	1.06–1.32	9.0	1.09	0.97–1.22	11.2	1.02	0.79–1.31
6.3–10.8%	4.4	1.26	1.09–1.46	8.8	1.15	1.00–1.32	10.6	1.06	0.79–1.43
<6.3%	4.2	1.36	1.14–1.61	8.6	1.22	1.02–1.46	10.1	1.05	0.73–1.51
Median Household Income Quartiles of Patients in Zip Code 2012–2016 ‡									
<$40,227	4.6	Ref.		8.9	Ref.		11.7	Ref.	
$40,227–50,353	4.6	0.91	0.81–1.02	8.9	0.96	0.85–1.08	11.4	0.97	0.74–1.26
$50,354–63,332	4.4	0.84	0.73–0.96	8.3	0.85	0.74–0.98	10.1	0.81	0.60–1.09
≥$63,333	3.9	0.65	0.54–0.78	7.8	0.73	0.61–0.87	9.9	0.76	0.53–1.09
Patient State at Diagnosis Grouped by Medicaid Expansion Status 2010–2016 ▲									
Non-Expansion States	4.0	Ref		9.5	Ref		10.3	Ref	
January 2014 Expansion States	5.0	1.26	1.04–1.54	10.8	1.23	1.04–1.45	12.0	1.21	0.92–1.61
Early Expansion States (2010–2013)	2.8	0.71	0.54–0.94	5.3	0.64	0.49–0.84	6.6	0.66	0.47–0.93
Late Expansion States (after January. 2014)	6.0	1.43	1.13–1.81	11.3	1.12	0.92–1.38	14.3	1.57	1.14–2.15
Greatest Circle Distance from Provider to Patient§									
<2 miles	5.6	Ref		10.5	Ref		12.4	Ref	
2–4 miles	5.3	0.97	0.84–1.12	9.0	0.82	0.71–0.95	9.8	0.81	0.59–1.12
5–9 miles	4.8	0.89	0.79–1.02	8.4	0.81	0.70–0.95	11.4	0.91	0.68–1.23
10–19 miles	4.3	0.85	0.73–0.98	8.3	0.79	0.68–0.92	10.6	0.88	0.65–1.21
20–45 miles	3.5	0.70	0.60–0.81	8.0	0.76	0.65–0.89	10.5	0.89	0.63–1.27
>45 miles	2.9	0.58	0.48–0.70	7.6	0.74	0.60–0.88	9.3	0.71	0.48–1.04
Facility Type ¶									
Community Cancer Program	6.6	1.18	0.95–1.45	10.3	1.01	0.81–1.27	13.1	1.10	0.81–1.49
Comprehensive Community Cancer Program	4.9	Ref		9.3	Ref		11.4	Ref	
Academic/Research Program	3.5	0.70	0.58–0.84	8.7	0.89	0.76–1.04	9.6	0.81	0.62–1.04
Integrated Network Cancer Program	4.5	0.90	0.73–1.10	11.2	1.18	1.00–1.39	10.3	0.85	0.63–1.15

Abbreviations: aOR: Adjusted odds ratio; CI: Confidence Interval; Ref.: Reference. * Adjusted for age, race/ethnicity, Charlson-Deyo comorbidity score, and median household income quartile of patient’s zip code. † Adjusted for age, race/ethnicity, census region, and median household income quartile of patient’s zip code. ‡ Adjusted for age, race/ethnicity, census region, and % of high school degree in quartile of patient’s zip code. ▲ Data were restricted to 2011–2016 as the Affordable Care Act was passed in 2010 and enacted in the following year (n = 88,637); Adjusted for age, Charlson-Deyo comorbidity score, and race/ethnicity. §Adjusted for age, race/ethnicity, area of residence and census region. ¶ Adjusted insurance type, area of residence, census region, and greatest circle distance to care.

## Data Availability

The data are publicly available by application through the American College of Surgeons at the following link: https://www.facs.org/quality-programs/cancer/ncdb/publicaccess. (accessed on 1 April 2021).

## Supplementary Materials

The following are available online at https://www.mdpi.com/article/10.3390/ijerph18116040/s1, Table S1: Associations of Health Care Access Factors with Palliative Care Use Among Deceased Metastatic Gynecological Cancer Patients by Cancer Site; Table S2: Associations of Health Care Access Measures with Palliative Care Receipt Among All Metastatic Gynecological Cancer Patients by Race/Ethnicity; Table S3: Associations of Health Care Access Indicators with Palliative Care Receipt Among Deceased Metastatic Gynecological Cancer Patients by Race/Ethnicity.

## References

[1] Dans M., Smith T., Back A., Baker J.N., Bauman J.R., Beck A.C., Block S., Campbell T., Case A.A., Dalal S. (2017). NCCN Guidelines Insights: Palliative Care, Version 2.2017. J. Natl. Compr. Cancer Netw..

[2] Kassianos A.P., Ioannou M., Koutsantoni M., Charalambous H. (2018). The impact of specialized palliative care on cancer patients’ health-related quality of life: A systematic review and meta-analysis. Support. Care Cancer.

[3] Bakitas M.A., Tosteson T.D., Li Z., Lyons K.D., Hull J.G., Li Z., Dionne-Odom J.N., Frost J., Dragnev K.H., Hegel M.T. (2015). Early Versus Delayed Initiation of Concurrent Palliative Oncology Care: Patient Outcomes in the ENABLE III Randomized Controlled Trial. J. Clin. Oncol..

[4] Nevadunsky N.S., Gordon S., Spoozak L., Van Arsdale A., Hou Y., Klobocista M., Eti S., Rapkin B., Goldberg G.L. (2014). The role and timing of palliative medicine consultation for women with gynecologic malignancies: Association with end of life interventions and direct hospital costs. Gynecol. Oncol..

[5] Rugno F.C., Paiva B.S., Paiva C.E. (2014). Early integration of palliative care facilitates the discontinuation of anticancer treatment in women with advanced breast or gynecologic cancers. Gynecol. Oncol..

[6] Bakitas M., Lyons K.D., Hegel M.T., Balan S., Brokaw F.C., Seville J., Hull J.G., Li Z., Tosteson T.D., Byock I.R. (2009). Effects of a palliative care intervention on clinical outcomes in patients with advanced cancer: The Project ENABLE II randomized controlled trial. JAMA.

[7] Greer J.A., Pirl W.F., Jackson V.A., Muzikansky A., Lennes I.T., Heist R.S., Gallagher E.R., Temel J.S. (2012). Effect of early palliative care on chemotherapy use and end-of-life care in patients with metastatic non-small-cell lung cancer. J. Clin. Oncol..

[8] Zimmermann C., Swami N., Krzyzanowska M., Hannon B., Leighl N., Oza A., Moore M., Rydall A., Rodin G., Tannock I. (2014). Early palliative care for patients with advanced cancer: A cluster-randomised controlled trial. Lancet.

[9] Ferrell B.R., Temel J.S., Temin S., Alesi E.R., Balboni T.A., Basch E.M., Firn J.I., Paice J.A., Peppercorn J.M., Phillips T. (2017). Integration of Palliative Care Into Standard Oncology Care: American Society of Clinical Oncology Clinical Practice Guideline Update. J. Clin. Oncol..

[10] Bauman J.R., Temel J.S. (2014). The integration of early palliative care with oncology care: The time has come for a new tradition. J. Natl. Compr. Cancer Netw..

[11] Kim Y.J., Munsell M.F., Park J.C., Meyer L.A., Sun C.C., Brown A.J., Bodurka D.C., Williams J.L., Chase D.M., Bruera E. (2015). Retrospective review of symptoms and palliative care interventions in women with advanced cervical cancer. Gynecol. Oncol..

[12] Deshields T.L., Potter P., Olsen S., Liu J. (2014). The persistence of symptom burden: Symptom experience and quality of life of cancer patients across one year. Support. Care Cancer.

[13] Nitecki R., Diver E.J., Kamdar M.M., Boruta D.M., Del Carmen M.C., Clark R.M., Goodman A., Schorge J.O., Growdon W.B. (2018). Patterns of palliative care referral in ovarian cancer: A single institution 5 year retrospective analysis. Gynecol. Oncol..

[14] Islam J.Y., Deveaux A., Previs R.A., Akinyemiju T. (2021). Racial and ethnic disparities in palliative care utilization among gynecological cancer patients. Gynecol. Oncol..

[15] Lopez-Acevedo M., Lowery W.J., Lowery A.W., Lee P.S., Havrilesky L.J. (2013). Palliative and hospice care in gynecologic cancer: A review. Gynecol. Oncol..

[16] Taylor J.S., Brown A.J., Prescott L.S., Sun C.C., Ramondetta L.M., Bodurka D.C. (2016). Dying well: How equal is end of life care among gynecologic oncology patients?. Gynecol. Oncol..

[17] Sheu J., Palileo A., Chen M.Y., Hoepner L., Abulafia O., Kanis M.J., Lee Y.C. (2019). Hospice utilization in advanced cervical malignancies: An analysis of the National Inpatient Sample. Gynecol. Oncol..

[18] Rosenfeld E.B., Chan J.K., Gardner A.B., Curry N., Delic L., Kapp D.S. (2018). Disparities Associated With Inpatient Palliative Care Utilization by Patients With Metastatic Gynecologic Cancers: A Study of 3337 Women. Am. J. Hosp. Palliat. Care.

[19] Islam J.Y., Deveaux A., Previs R.A., Akinyemiju T. (2021). Racial disparities in palliative care utilization among metastatic gynecological cancer patients living at last follow-up: An analysis of the National Cancer Data Base. Data Brief.

[20] Bilimoria K.Y., Stewart A.K., Winchester D.P., Ko C.Y. (2008). The National Cancer Data Base: A powerful initiative to improve cancer care in the United States. Ann. Surg. Oncol..

[21] Boffa D.J., Rosen J.E., Mallin K., Loomis A., Gay G., Palis B., Thoburn K., Gress D., McKellar D.P., Shulman L.N. (2017). Using the National Cancer Database for Outcomes Research: A Review. JAMA Oncol..

[22] McCabe R.M. (2019). National Cancer Database: The Past, Present, and Future of the Cancer Registry and Its Efforts to Improve the Quality of Cancer Care. Semin. Radiat. Oncol..

[23] CTR Exam. https://www.ncra-usa.org/CTR/Certification-Exam.

[24] Winchester D.P., Stewart A.K., Phillips J.L., Ward E.E. (2010). The national cancer data base: Past, present, and future. Ann. Surg. Oncol..

[25] Cole A.P., Nguyen D.D., Meirkhanov A., Golshan M., Melnitchouk N., Lipsitz S.R., Kilbridge K.L., Kibel A.S., Cooper Z., Weissman J. (2019). Association of Care at Minority-Serving vs Non-Minority-Serving Hospitals With Use of Palliative Care Among Racial/Ethnic Minorities With Metastatic Cancer in the United States. JAMA Netw. Open.

[26] Haque W., Verma V., Butler E.B., Teh B.S. (2019). Patterns of End-of-Life Oncologic Care for Stage IV Non-small Cell Lung Cancer in the United States. Anticancer Res..

[27] Colibaseanu D.T., Osagiede O., Spaulding A.C., Frank R.D., Merchea A., Mathis K.L., Parker A.S., Ailawadhi S. (2018). The Determinants of Palliative Care Use in Patients With Colorectal Cancer: A National Study. Am. J. Hosp. Palliat. Care.

[28] National Cancer Data Base Participant User File (PUF) Data Dictionary. https://www.facs.org/-/media/files/quality-programs/cancer/ncdb/puf_data_dictionary.ashx.

[29] Medicaid.gov-Keeping America Healthy. https://www.medicaid.gov/medicaid/index.html.

[30] What’s Medicare?. https://www.medicare.gov/what-medicare-covers/your-medicare-coverage-choices/whats-medicare.

[31] Medicaid Expansion. https://www.healthinsurance.org/glossary/medicaid-expansion/.

[32] Takvorian S.U., Oganisian A., Mamtani R., Mitra N., Shulman L.N., Bekelman J.E., Werner R.M. (2020). Association of Medicaid Expansion Under the Affordable Care Act With Insurance Status, Cancer Stage, and Timely Treatment Among Patients With Breast, Colon, and Lung Cancer. JAMA Netw. Open.

[33] American Community Survey (ACS). https://www.census.gov/programs-surveys/acs.

[34] About Race. https://www.census.gov/topics/population/race/about.html.

[35] About Hispanic Origin. https://www.census.gov/topics/population/hispanic-origin/about.html.

[36] Ambroggi M., Biasini C., Del Giovane C., Fornari F., Cavanna L. (2015). Distance as a Barrier to Cancer Diagnosis and Treatment: Review of the Literature. Oncologist.

[37] Spees L.P., Brewster W.R., Varia M.A., Weinberger M., Baggett C., Zhou X., Petermann V.M., Wheeler S.B. (2019). Examining Urban and Rural Differences in How Distance to Care Influences the Initiation and Completion of Treatment among Insured Cervical Cancer Patients. Cancer Epidemiol. Biomark. Prev..

[38] Lin C.C., Bruinooge S.S., Kirkwood M.K., Olsen C., Jemal A., Bajorin D., Giordano S.H., Goldstein M., Guadagnolo B.A., Kosty M. (2015). Association Between Geographic Access to Cancer Care, Insurance, and Receipt of Chemotherapy: Geographic Distribution of Oncologists and Travel Distance. J. Clin. Oncol. Off. J. Am. Soc. Clin. Oncol..

[39] Ricci S., Tergas A.I., Roche K.L., Fairbairn M.G., Levinson K.L., Dowdy S.C., Bristow R.E., Lopez M., Slaughter K., Moore K. (2017). Geographic disparities in the distribution of the U.S. gynecologic oncology workforce: A Society of Gynecologic Oncology study. Gynecol. Oncol. Rep..

[40] Huff C. (2019). Bringing Palliative Care To Underserved Rural Communities. Health Aff..

[41] Osagiede O., Colibaseanu D.T., Spaulding A.C., Frank R.D., Merchea A., Kelley S.R., Uitti R.J., Ailawadhi S. (2018). Palliative Care Use Among Patients With Solid Cancer Tumors: A National Cancer Data Base Study. J. Palliat. Care.

[42] Morrison R.S., Dietrich J., Ladwig S., Quill T., Sacco J., Tangeman J., Meier D.E. (2011). Palliative care consultation teams cut hospital costs for Medicaid beneficiaries. Health Aff..

[43] Morrison R.S. (2013). Models of palliative care delivery in the United States. Curr. Opin. Support. Palliat. Care.

[44] Morrison R.S., Penrod J.D., Cassel J.B., Caust-Ellenbogen M., Litke A., Spragens L., Meier D.E. (2008). Cost savings associated with US hospital palliative care consultation programs. Arch. Intern. Med..

[45] Goldsmith B., Dietrich J., Du Q., Morrison R.S. (2008). Variability in access to hospital palliative care in the United States. J. Palliat. Med..

[46] Washington K.T., Bickel-Swenson D., Stephens N. (2008). Barriers to hospice use among African Americans: A systematic review. Health Soc. Work.

[47] Soobader M., LeClere F.B., Hadden W., Maury B. (2001). Using aggregate geographic data to proxy individual socioeconomic status: Does size matter?. Am. J. Public Health.

