# Supplementary material for: Health Care Access Measures and Palliative Care Use by Race/Ethnicity among Metastatic Gynecological Cancer Patients in the United States
Source: Int J Environ Res Public Health. 2021 Jun 4;18(11):6040. doi: 10.3390/ijerph18116040 (PMC8200023; doi:10.3390/ijerph18116040)
Supplement: Supplementary file 1 [file ijerph-18-06040-s001.zip › ijerph-1213315-supplementary.pdf]

Table S1. Associations of Health Care Access Factors with Palliative Care Use Among Deceased Metastatic Gynecological Cancer Patients by Cancer Site.

	Ovarian Cancer (n= 91969)			Cervical Cancer (n=27435)			Uterine Cancer (n=5325)		
	% Utilized Palliative Care	aOR	95% CI	% Utilized Palliative Care	aOR	95% CI	% Utilized Palliative Care	aOR	95% CI
Primary Payer*									
Not Insured	5.0	1.62	1.35-1.94	12.1	1.41	1.21-1.64	13.5	1.15	0.78-1.68
Private Insurance/Managed Care	3.4	Ref.		9.4	Ref.		11.8	Ref.	
Medicaid	6.0	1.79	1.53-2.09	11.6	1.34	1.19-1.51	13.2	1.18	0.86-1.62
Medicare	6.5	1.19	1.08-1.32	14.4	1.12	1.00-1.26	12.2	1.04	0.82-1.32
Other Government	5.4	1.60	1.12-2.27	11.1	1.17	0.77-1.79	7.1	0.72	0.21-2.42
Insurance Status Unknown	4.2	1.06	0.76-1.48	9.1	1.03	0.76-1.39	12.8	1.25	0.70-2.24
Percent of Adults in Patient Zip Code with No High School Degree Quartiles 2012-2016†									
>=17.6%	4.8	Ref.		10.6	Ref.		12.1	Ref.	
10.9-17.5%	5.5	1.15	1.01-1.30	12.2	1.10	0.98-1.25	13.1	1.04	0.79-1.36
6.3-10.8%	5.3	1.24	1.06-1.44	12.3	1.17	1.01-1.35	12.4	1.09	0.79-1.49
<6.3%	5.3	1.35	1.13-1.62	11.9	1.24	1.02-1.50	11.6	1.05	0.71-1.54
Median Household Income Quartiles of Patients in Zip Code 2012-2016‡									
< $40,227	5.4	Ref.		11.8	Ref.		13.2	Ref.	
$40,227-50,353	5.5	0.94	0.83-1.05	12.0	0.95	0.84-1.09	13.4	0.98	0.75-1.30
$50,354-63,332	5.5	0.88	0.77-1.01	11.7	0.89	0.77-1.03	11.3	0.79	0.58-1.08
>=$63,333	4.9	0.69	0.57-0.83	11.0	0.76	0.63-0.92	11.7	0.77	0.54-1.12
Patient State at Diagnosis Grouped by Medicaid Expansion Status 2010-2016▲									
Non-Expansion States	6.4	Ref		14.1	Ref		13.3	Ref	
January 2014 Expansion States	8.2	1.28	1.05-1.56	17.4	1.26	1.05-1.51	16.3	1.27	0.95-1.70
Early Expansion States (2010-2013)	4.7	0.71	0.53-0.94	9.9	0.68	0.51-0.90	9.2	0.64	0.46-0.93
Late Expansion States (after Jan. 2014)	9.0	1.44	1.13-1.83	15.6	1.10	0.88-1.37	19.8	1.65	1.19-2.29
Greatest Circle Distance from Provider to Patient§									
<2 miles	6.7	Ref		13.3	Ref		14.4	Ref	
2-4 miles	6.4	0.98	0.85-1.14	12.1	0.86	0.73-1.02	11.3	0.82	0.58-1.15
5-9 miles	5.8	0.91	0.79-1.04	11.6	0.87	0.74-1.02	13.1	0.92	0.67-1.25
10-19 miles	5.3	0.86	0.74-0.99	11.8	0.86	0.74-1.01	11.9	0.85	0.61-1.17
20-45 miles	4.3	0.71	0.60-0.83	11.0	0.81	0.68-0.96	12.3	0.89	0.62-1.28
>45 miles	3.6	0.59	0.49-0.72	10.7	0.79	0.65-0.97	10.7	0.71	0.48-1.07
Facility Type¶									
Community Cancer Program	7.7	1.18	0.95-1.46	12.9	0.99	0.79-1.24	14.8	1.08	0.79-1.48
Comprehensive Community Cancer Program	5.8	Ref		12.3	Ref		13.0	Ref	
Academic/Research Program	4.3	0.73	0.60-0.88	11.6	0.90	0.76-1.06	11.1	0.79	0.61-1.04
Integrated Network Cancer Program	5.5	0.92	0.75-1.13	15.0	1.21	1.02-1.45	12.1	0.86	0.62-1.18

*Adjusted for age, race/ethnicity, Charlson-Deyo comorbidity score, and median household income quartile of patient's zip code
†Adjusted for age, race/ethnicity, census region, and median household income quartile of patient's zip code
‡Adjusted for age, race/ethnicity, census region, and % of high school degree in quartile of patient's zip code
▲Data were restricted to 2011-2016 as the Affordable Care Act was passed in 2010 and enacted in the following year (n=55,871); Adjusted for age, Charlson-Deyo comorbidity score, and race/ethnicity

§Adjusted for age, race/ethnicity, area of residence and census region
¶Adjusted insurance type, area of residence, census region, and greatest circle distance to care

Table S2. Associations of Health Care Access Measures with Palliative Care Receipt Among All Metastatic Gynecological Cancer Patients by Race/Ethnicity.

	NH-White			NH-Black			Hispanic			Asian		
	aOR	95% CI		aOR	95% CI		aOR	95% CI		aOR	95% CI	
Primary Payer *												
Not Insured	1.98	1.74	2.24	1.47	1.18	1.83	1.86	1.38	2.51	1.06	0.59	1.89
Private Insurance/Managed Care	Ref			Ref			Ref					
Medicaid	2.40	2.16	2.66	1.38	1.14	1.67	1.29	0.95	1.76	1.37	0.90	2.10
Medicare	1.18	1.09	1.28	1.25	1.06	1.48	1.12	0.85	1.47	1.14	0.77	1.68
Other Government	1.42	1.09	1.85	1.36	0.68	2.73	0.98	0.29	3.24	-	-	-
Percent of Adults in Patient Zip Code with No High School Degree Quartiles 2012-2016†												
>=17.6%	Ref			Ref			Ref			Ref.		
10.9-17.5%	1.10	0.99	1.23	0.90	0.78	1.03	1.18	0.88	1.59	1.54	0.97	2.43
6.3-10.8%	1.16	0.98	1.27	0.95	0.77	1.18	1.16	0.81	1.66	2.15	1.32	3.51
<6.3%	1.15	0.99	1.34	1.04	0.76	1.42	1.12	0.72	1.74	2.41	1.43	4.07
Median Household Income Quartiles of Patients in Zip Code 2012-2016‡												
< $40,227	Ref			Ref			Ref			Ref.		
$40,227-50,353	0.89	0.80	0.98	1.11	0.93	1.32	0.76	0.58	0.99	1.04	0.54	2.00
$50,354-63,332	0.79	0.70	0.89	1.03	0.83	1.29	0.70	0.52	0.94	0.86	0.45	1.63
>=$63,333	0.62	0.52	0.73	0.89	0.68	1.16	0.67	0.47	0.95	0.56	0.28	1.10
Patient State at Diagnosis Grouped by Medicaid Expansion Status 2010-2016▲												
Non-Expansion States	Ref			Ref			Ref			Ref.		
January 2014 Expansion States	1.27	1.07	1.49	1.14	0.91	1.43	1.14	0.97	1.92	1.18	0.65	2.14
Early Expansion States (2010-2013)	0.67	0.53	0.84	0.86	0.61	1.21	0.61	0.40	0.93	0.66	0.36	1.20
Late Expansion States (after Jan. 2014)	1.39	1.15	1.69	1.02	0.73	1.43	2.25	1.33	3.79	0.99	0.46	2.17
Greatest Circle Distance from Provider to Patient§												
<2 miles	Ref			Ref			Ref			Ref.		
2-4 miles	0.93	0.82	1.06	0.82	0.66	1.01	0.97	0.71	1.32	0.67	0.42	1.07
5-9 miles	0.89	0.79	1.00	0.75	0.60	0.93	0.90	0.64	1.26	0.54	0.35	0.83
10-19 miles	0.83	0.73	0.94	0.72	0.57	0.91	0.90	0.64	1.27	0.68	0.39	1.19
20-45 miles	0.73	0.64	0.84	0.67	0.51	0.89	0.91	0.62	1.34	0.61	0.33	1.15
>45 miles	0.63	0.53	0.74	0.46	0.35	0.61	0.71	0.45	1.13	0.34	0.14	0.83
Facility Type¶												
Community Cancer Program	1.23	1.01	1.49	1.03	0.71	1.50	0.71	0.44	1.15	0.88	0.37	2.10
Comprehensive Community Cancer Program	Ref			Ref			Ref			Ref.		
Academic/Research Program	0.73	0.62	0.86	0.95	0.76	1.20	0.91	0.65	1.27	1.38	0.74	2.57
Integrated Network Cancer Program	0.92	0.77	1.10	1.12	0.86	1.46	1.14	0.75	1.72	2.01	1.15	3.51

*Adjusted for age, Charlson-Deyo comorbidity score, and median household income quartile of patient's zip code; Other government category was dropped (n=91) from the model evaluating Asian gynecological patients as no patients in this category received palliative care.
†Adjusted for age, census region, and median household income quartile of patient's zip code
‡Adjusted for age, census region, and % of high school degree in quartile of patient's zip code
▲ Data were restricted to 2011-2016 as the Affordable Care Act was passed in 2010 and enacted in the following year (n=88,637); Adjusted for age and Charlson-Deyo comorbidity score
§Adjusted for age, area of residence and census region; Rural area of residence was dropped from the model (n=5) evaluating Asian gynecological patients as no patients in this category received palliative care

¶Adjusted insurance type, area of residence, census region, and greatest circle distance to care; Other government category (n=78) and rural area (n= 5) were dropped from the model evaluating Asian gynecological patients as no patients in this category received palliative care.

Table S3. Associations of Health Care Access Indicators with Palliative Care Receipt Among Deceased Metastatic Gynecological Cancer Patients by Race/Ethnicity.

	**NH-White**			**NH-Black**			**Hispanic**			**Asian**		
	**aOR**	**95% CI**		**aOR**	**95% CI**		**aOR**	**95% CI**		**aOR**	**95% CI**	
Primary Payer*												
Not Insured	1.89	1.65	2.16	1.46	1.13	1.90	1.63	1.18	2.24	1.26	0.67	2.39
Private Insurance/Managed Care	Ref			Ref			Ref			Ref.		
Medicaid	2.33	2.09	2.59	1.34	1.08	1.66	1.14	0.80	1.62	1.31	0.84	2.05
Medicare	1.19	1.10	1.30	1.28	1.06	1.54	0.96	0.70	1.33	1.21	0.8	1.83
Other Government	1.41	1.06	1.87	1.58	0.81	3.10	1.30	0.39	4.36	-		
Insurance Status Unknown	1.12	0.83	1.50	1.09	0.65	1.83	1.18	0.61	2.27	1.8	0.66	4.85
Percent of Adults in Patient Zip Code with No High School Degree Quartiles 2012-2016†												
>=17.6%	Ref			Ref			Ref			Ref.		
10.9-17.5%	1.10	0.99	1.23	0.88	0.76	1.02	1.12	0.79	1.58	1.25	0.74	2.10
6.3-10.8%	1.11	0.98	1.26	0.98	0.78	1.22	1.08	0.72	1.60	2.22	1.32	3.73
<6.3%	1.15	0.98	1.34	1.13	0.81	1.57	1.19	0.72	1.96	2.32	1.32	4.07
Median Household Income Quartiles of Patients in Zip Code 2012-2016‡												
< $40,227	Ref			Ref			Ref			Ref.		
$40,227-50,353	0.88	0.79	0.98	1.12	0.93	1.36	0.77	0.56	1.06	1.52	0.68	3.51
$50,354-63,332	0.80	0.71	0.91	1.02	0.81	1.29	0.84	0.60	1.17	1.32	0.59	2.99
>=$63,333	0.65	0.55	0.76	0.88	0.66	1.17	0.74	0.50	1.10	0.78	0.33	1.78
Patient State at Diagnosis Grouped by Medicaid Expansion Status 2010-2016▲												
Non-Expansion States	Ref			Ref			Ref			Ref		
January 2014 Expansion States	1.29	1.09	1.52	1.11	0.87	1.41	1.57	1.08	2.30	1.07	0.58	1.97
Early Expansion States (2010-2013)	0.69	0.55	0.87	0.88	0.61	1.25	0.60	0.37	0.96	0.59	0.32	1.08
Late Expansion States (after Jan. 2014)	1.40	1.14	1.70	1.01	0.74	1.39	2.34	1.13	4.37	0.99	0.43	2.28
Greatest Circle Distance from Provider to Patient§												
<2 miles	Ref			Ref			Ref			Ref.		
2-4 miles	0.94	0.83	1.08	0.86	0.69	1.07	1.04	0.71	1.52	0.77	0.48	1.24
5-9 miles	0.90	0.80	1.02	0.79	0.63	0.98	1.04	0.71	1.51	0.52	0.31	0.87
10-19 miles	0.86	0.75	0.97	0.73	0.57	0.94	0.83	0.55	1.25	0.75	0.43	1.32
20-45 miles	0.75	0.65	0.86	0.72	0.53	0.97	0.94	0.61	1.45	0.44	0.21	1.02
>45 miles	0.65	0.55	0.78	0.47	0.35	0.63	0.74	0.44	1.27	0.33	0.13	0.80
Facility Type¶												
Community Cancer Program	1.20	0.98	1.46	1.03	0.71	1.51	0.82	0.50	1.35	1.04	0.47	2.31
Comprehensive Community Cancer Program	Ref			Ref			Ref			Ref.		
Academic/Research Program	0.75	0.63	0.89	0.96	0.75	1.21	1.03	0.71	1.50	1.45	0.75	2.79
Integrated Network Cancer Program	0.94	0.79	1.13	1.11	0.85	1.46	1.28	0.82	2.00	2.13	1.22	3.73

*Adjusted for age, Charlson-Deyo comorbidity score, and median household income quartile of patient's zip code; Other government category was dropped (n=39) from the model evaluating Asian gynecological patients as no patients in this category received palliative care.

†Adjusted for age, census region, and median household income quartile of patient's zip code

‡Adjusted for age, census region, and % of high school degree in quartile of patient's zip code

▲ Data were restricted to 2011-2016 as the Affordable Care Act was passed in 2010 and enacted in the following year (n=55,871); Adjusted for age, and Charlson-Deyo comorbidity score

§Adjusted for age, area of residence and census region; Rural area of residence was dropped from the model (n=4) evaluating Asian gynecological patients as no patients in this category received palliative care

¶ Adjusted insurance type, area of residence, census region, and greatest circle distance to care; Other government category (n=39) and rural area (n= 4) were dropped from the model evaluating Asian gynecological patients as no patients in this category received palliative care.
